# Development of the Arabic Voice Pathology Database and Its Evaluation by Using Speech Features and Machine Learning Algorithms

**DOI:** 10.1155/2017/8783751

**Published:** 2017-10-19

**Authors:** Tamer A. Mesallam, Mohamed Farahat, Khalid H. Malki, Mansour Alsulaiman, Zulfiqar Ali, Ahmed Al-nasheri, Ghulam Muhammad

**Affiliations:** ^1^ENT Department, College of Medicine, King Saud University, Riyadh, Saudi Arabia; ^2^Digital Speech Processing Group, Department of Computer Engineering, College of Computer and Information Sciences, King Saud University, Riyadh, Saudi Arabia

## Abstract

A voice disorder database is an essential element in doing research on automatic voice disorder detection and classification. Ethnicity affects the voice characteristics of a person, and so it is necessary to develop a database by collecting the voice samples of the targeted ethnic group. This will enhance the chances of arriving at a global solution for the accurate and reliable diagnosis of voice disorders by understanding the characteristics of a local group. Motivated by such idea, an Arabic voice pathology database (AVPD) is designed and developed in this study by recording three vowels, running speech, and isolated words. For each recorded samples, the perceptual severity is also provided which is a unique aspect of the AVPD. During the development of the AVPD, the shortcomings of different voice disorder databases were identified so that they could be avoided in the AVPD. In addition, the AVPD is evaluated by using six different types of speech features and four types of machine learning algorithms. The results of detection and classification of voice disorders obtained with the sustained vowel and the running speech are also compared with the results of an English-language disorder database, the Massachusetts Eye and Ear Infirmary (MEEI) database.

## 1. Introduction

The Arabic voice pathology database (AVPD) will have a potential impact on the assessment of voice disorders in the Arab region. Race has been suggested to contribute to the perception of voice, with Walton and Orlikoff [1] showing, for example, that measures of amplitude and frequency perturbation in African-American adult males are not equal to those of white adult males. Additionally, Sapienza [2] analyzed the vowel /a/ in a group of 20 African Americans and 20 white Americans, finding that African-American males and females had higher mean fundamental frequencies and lower sound pressure levels, although the differences were not significant. This difference was partially attributed to the large ratio of the membranous to cartilaginous portion of the vocal folds and increased thickness, a finding previously reported by Boshoff [3]. Sapienza [2] did not examine other acoustic parameters for gender or racial differences. Walton and Orlikoff [1] found, through acoustical analysis, that African-American speakers had significantly greater amplitude perturbation measures and significantly lower harmonics-to-noise ratios than did white adult males. Although the former had a lower mean speaking fundamental frequency than the latter, the differences were not significant in the group of 50 subjects.

In a study by Malki et al. [4], the acoustic voice analysis of 100 normal Saudi adult subjects was compared using built-in normative data in the KayPENTAX Multi-Dimensional Voice Program (MDVP) software [5]. The authors concluded that fundamental frequency and many of the frequency and amplitude perturbation variables showed statistically significant differences between normal Saudi males and females. In addition, the groups of Saudi males and females showed significant differences in the perturbation parameters compared with a standard North American database. The data from this study support the hypothesis that these differences are most likely due to racial differences. This is the reason that researchers have used databases of different languages in the studies to investigate the findings more precisely. A Korean database is used in [6], a German one is considered in [7], another German database is used in [8], a Spanish database is considered in [9], and another Spanish database is used in [10].

Voice disorder databases can be used in clinics as well as in automatic voice disorder detection systems to study the acoustic behavior of the voices suffering from different types of vocal disorders. The evaluation of disordered speech, such as dysphonia, is a vital element in the clinical appraisal and treatment of the human voice. In addition to endoscopic examination of the larynx and vocal folds, perceptual and acoustic measurement techniques are crucial components of the clinical assessment of dysphonia. The perceptual assessment includes various rating scales such as the consensus auditory perceptual evaluation of voice (CAPE-V) scale [11] and the Grade, Roughness, Breathiness, Asthenia, Strain (GRBAS) scale [12] that assesses the overall grade of dysphonia, degree of roughness, breathiness, asthenia, and strain. Although those techniques are commonly used in clinical practice, there are potential limitations for their application because of the subjective nature of the evaluation. Those limitations may include the clinician's experience, the degree of the patient's dysphonia, the type of auditory perceptual rating scale, and the stimulus or speaking task. Based on the pitfalls of the perceptual evaluation, clinicians and researchers have developed a more objective tool for quantifying the degree of dysphonia that patients have via the acoustic analysis of voice. As a result of acoustic analysis, a numerical value is obtained that describes the severity of the pathology, allows for treatment and follow-up, and makes this information available to other stakeholders.

A database should contain a variety of recorded text, as many voice clinicians use sustained vowel samples rather than continuous speech samples in performing an acoustic analysis of their patients. Although some researchers have found that the sustained vowel is optimal for obtaining a voice sample for a variety of reasons, it does not truly represent voice use patterns in daily speech. At the same time, fluctuations of vocal characteristics in relation to voice onset, voice termination, and voice breaks, which are considered to be crucial in voice quality evaluation, are not fully represented in short signals of phonation such as sustained vowels. Furthermore, dysphonic symptoms are often more evident in conversational voice production than sustained vowels, and they are most often gestured by the dysphonic persons themselves in continuous speech. In addition, some voice pathologies, like adductor spasmodic dysphonia during sustained vowel production, can be distinguished from a relatively normal voice. Moreover, some of the acoustic correlates of an individual's voice are the result of the influence of the segmental and suprasegmental structure of speech that cannot be represented in the sustained vowel.

A good quality voice disorder database can help to solve the growing number of voice complications in the Arab region and beyond. The number of patients with a voice pathology has increased significantly in recent years, with approximately 17.9 million people in the United States alone suffering from a vocal difficulty [13]. It has been found that 15% of the total visitors to the King Abdulaziz University Hospital in Saudi Arabia complain of a voice disorder [14]. The complications caused by a voice problem in a teaching professional are significantly greater than in a nonteaching professional, and studies have revealed that, in the United States, the prevalence of voice disorders during a person's lifetime is 57.7% for teachers and 28.8% for nonteachers [15]. Approximately 33% of male and female teachers in the Riyadh area of Saudi Arabia suffer from voice disorders [16]. However, spasmodic dysphonia is a voice disorder caused by involuntary movements of the muscles of the larynx. At our voice center at the Communication and Swallowing Disorders Unit of the King Abdulaziz University Hospital, we see a high volume of voice disorder cases (almost 760 cases per year) in individuals with various professional and etiological backgrounds.

Based on our previous study [4], which explored the acoustic voice characteristics of normal adult Saudi subjects and a voice sample database derived from normal North American subjects, there were significant differences between the two groups when only a sustained vowel was analyzed. Accordingly, we will study the acoustic correlates in the voice-disordered Arab population and use the developed AVPD to explore the acoustic characteristics of the voices in comparison with other databases, especially when incorporating connected speech in the analysis.

The recorded AVPD database is evaluated by using many speech features to provide baseline results. The features are MDVP, Mel-frequency Cepstral Coefficients (MFCC) [17, 18], Linear Predictive Cepstral Coefficients (LPCC) [19], Linear Prediction Coefficients (LPC) [20], Perceptual Linear Predictive Coefficients [21], and Relative Spectral Transform-Perceptual Linear Prediction Coefficients (RASTA-PLP) [22]. To generate the acoustic models of normal and different types of voice disorder, a number of machine learning algorithms are implemented with each type of speech feature. The algorithms are Gaussian Mixture Model (GMM) [23], Hidden Markov Model (HMM) [24, 25], Support Vector Machine (SVM) [26], and Vector Quantization (VQ) based on the Linde-Buzo-Gray algorithm [27].

The rest of the paper is organized as follows.  Section 2 describes the design and development of the AVPD database, Section 3 delivers the baseline results of the AVPD by using various speech features and machine learning algorithms, Section 4 provides the discussion, and Section 5 draws some conclusions.

## 2. Arabic Voice Pathology Database

This section describes the steps to designing and developing the AVPD and includes an overview of the text recorded and provides the statistics of the database. Moreover, segmentation and verification processes are also discussed.

### 2.1. Video-Laryngeal Stroboscopic Examination

KayPENTAX's video-laryngeal stroboscopic system (Model 9200C2) was used in the examination, including a 70° rigid endoscope, 3CCD Toshiba camera, Sony LCD monitor, and a light source (Model RLS 9100B). Clinical diagnosis and classification of voice disorders were decided based on laryngoscopic examination. Two experienced phoniatricians were responsible for clinical diagnosis and classification of voice disorders. In case of unclear diagnosis, two examiners reviewed the recorded video-laryngeal examinations and a consensus decision about clinical diagnosis was obtained.

### 2.2. Recording Equipment and Protocol

The AVPD recorded both normal and disordered subjects by using Computerized Speech Lab model 4500 (CSL 4500), a product of KayPENTAX (Montvale, NJ, USA). All subjects were recorded by expert clinicians in a sound treated room at the Communication and Swallowing Disorders Unit of King Abdulaziz University Hospital. The sampling frequency of the recorded samples was 48 kHz with a bit rate of 16 bits. All recordings were done by keeping a fixed distance of 15 cm between mouth and microphone and stored in two different audio formats. Five organic voice disorders, vocal fold cysts, nodules, paralysis, polyps, and sulcus, were considered in the AVPD. In addition, all normal subjects were recorded after clinical evaluation to make sure that they were not suffering from any voice disorder and also that they had not experienced a voice complication in the past. Information such as subject's gender, age, and smoking habit was also collected, and each subject signed a document to show their consent and to record that they did not have any objections to their recorded samples being used for research purposes. Moreover, perceptual severity of disordered voice quality was rated on a scale of 1 to 3, where 1 represents mild, 2 represents moderate, and 3 represents severe voice quality disorder. A variety of text was recorded by each subject in the AVPD, which is explained in the following subsection.

### 2.3. Recording Text

Three types of text, including three vowels, isolated words, and running speech, were considered during the development of the AVPD. The text was compiled in a way that ensured that it was simple and short, and at the same time it covered all the Arabic phonemes. The first type of text was three vowels, fatha 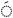 /a/, damma 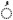 /u/, and kasra 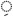 /i/, which were recorded with a repetition, including onset and offset information. The second type of text involved isolated words, including Arabic digits from zero to ten and some common words (see Tables 1 and 2). The third type of text was running speech (see Table 3), and the continuous speech was taken from the first chapter of the Quran, called the Al-Fateha.

The third type of text is running speech, and it is given in Table 3. The continuous speech is the first chapter from the Holy book of Muslims, called Al-Fateha. One of the reasons behind the selection of the religious text is that most of the visitors to our voice disorder unit are illiterate. Therefore, we selected the religious text because every Muslim memorizes it by heart. The other reason is the duration of Al-Fateha which is 20 seconds, and it is better than the duration of running speech of MEEI database (9 seconds) and SVD database (2 seconds).

The Arabic digits and Al-Fateha covered all the Arabic letters except three: ‎‎‎ج‎, ز‎, and ظ‎. Therefore, some common words were included in the text to cover these omissions. These words were ظرف (envelope), غزال (deer), and جمل (camel), as mentioned in Table 2. The number of occurrences of each Arabic letter in the recorded text is mentioned in Table 4. For illiterate patients, we have shown pictures of ظرف (envelope), غزال (deer), and جمل (camel) to record these words.

### 2.4. Statistics

**Figure 1:**
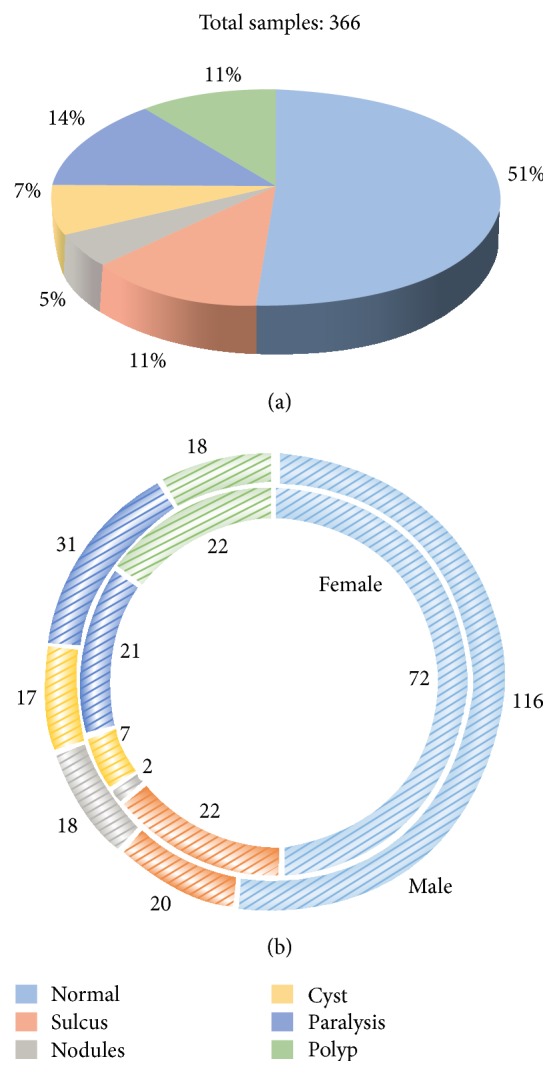
(a) Distribution of normal and voice disorder subjects in the AVPD. (b) Number of male and female samples for each disorder and normal subjects.

Overall, 366 samples of normal and pathological subjects are recorded in the AVPD. Normal subjects are 51% of the total subjects, and the remaining subjects are distributed among five voice disorders: sulcus 11%, nodules 5%, cyst 7%, paralysis 14%, and polyp 11% (Figure 1(a)). Among the 51% of normal subjects (188 samples), there are 116 male and 82 female speakers. In addition, the number of pathologic male and female patients, respectively, is as follows for the different disorders: sulcus 20 and 22, nodules 18 and 2, cysts 17 and 7, paralysis 31 and 21, and polyps 18 and 22 (Figure 1(b)). The inner ring in Figure 1(b) represents the number of female subjects, while the outer ring shows the number of male subjects.

**Figure 2:**
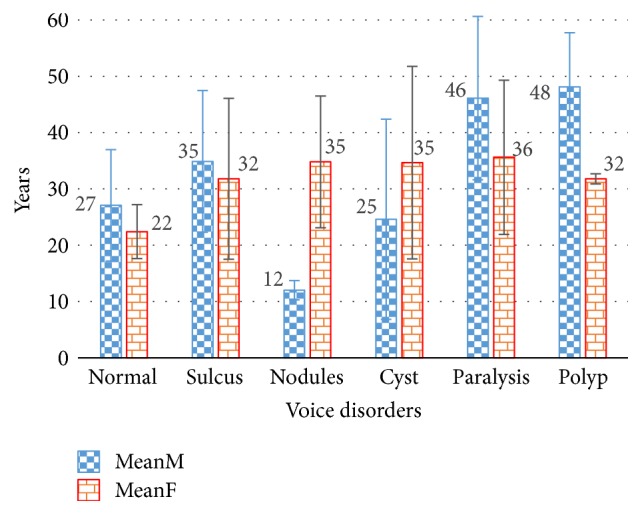
Age distribution of male and female subjects in the AVPD.

Approximately 60% of the subjects in the AVPD are male, while 40% are female. The information about the mean age (in years) of the recorded subjects with standard deviation (STD) is provided in Figure 2. The average age ± STD of male subjects who are normal or suffering from sulcus, nodules, cysts, paralysis, or polyps is 27 ± 10, 35 ± 13, 12 ± 2, 25 ± 18, 46 ± 15, and 48 ± 10 years, respectively, while for female subjects it is 22 ± 5, 32 ± 14, 35 ± 12, 35 ± 17, 36 ± 14, and 32 ± 10 years, respectively. A consent form is signed by each normal and disordered subject before recording of his∖her voice sample. In the consent form, each subject testified that his∖her participation is completely voluntary, and their decisions will not affect the medical care they receive.

### 2.5. Terminology for File Name

All the text, including three vowels with a repetition, Arabic digits, Al-Fateha, and common words, was recorded and stored in two different audio formats,* wav* and* nsp*. The file names consist of eight parts and are stored as follows:

  disorder-sex-age-smoke-code-severity-surgery.wav
  disorder-sex-age-smoke-code-severity-surgery.nsp

The first part of the file name is the type of voice disorder. Three-letter abbreviations are used to represent each voice disorder:* cyt* for cysts,* ndl* for nodules,* prl *for paralysis,* plp* for polyps, and* sls* for sulcus. The abbreviation used to represent nonpathological subjects is* nor*. The second part of the file name refers to the sex of the subject and so is either male* (M)* or female* (F)*. The third part is the age of the subject, while the fourth part denotes whether he or she is a smoker or not. If a subject is a smoker, then the file name includes* y*, but if they are not then it includes* n*. The fifth part is a unique alphanumeric code which is assigned to every subject to maintain their history. The sixth part is perceptual severity of the voice disorder, which is rated on a scale of 1 (mild) to 3 (severe), whereas perceptual severity of a normal subject is represented by 0. The seventh part provides the information that a sample is recorded before surgery or after surgery. The samples recorded before and after surgery are denoted by* p* and* t*, respectively. The last part denotes the extension of a file, which may be* wav* or* nsp*.

For instance, consider the file name* plp-m-20-y-15034kam-2-p.wav, *which indicates that a male smoker of age 20 years is suffering from a polyp. In addition, the file name indicates that perceptual severity is moderate and the sample was recorded before surgery. The file has been stored in* wav* format. Some voice samples recorded by a normal person and patients suffering from different disorders are provided as a Supplementary Material available online at https://doi.org/10.1155/2017/8783751. These supplementary samples are cyt-m-55-n-360527-kam-3-p.wav, nor-m-23-n-90021-kac-0-p.wav, prl-f-34-n-569624-kac-2-p.wav, and sls-m-20-y-545716-kac-1-p.wav.

### 2.6. Segmentation of Recorded Samples

Recorded samples were divided into the following 22 segments: six segments for vowels (three vowels plus their repetition), 11 segments for Arabic digits (zero to ten), two segments for Al-Fateha (divided in this manner so that the first part may be used to train the system and the second part to test the system), and three segments for the common words. The first part of Al-Fateha starts from sentence number 1 and ends at 4, while the second part contains the last three sentences.

Each of the 22 segments was stored in a separate* wav* file. The segmentation was performed with the help of Praat software [28] by labeling the start and end time of each segment. Then, these two times were used to extract a segment from a recorded sample. Once each recorded sample was divided into segments and stored into 22* wav* files, the next step was the verification process, which ensured that each segmented* wav* file consisted of a complete utterance. During the verification process, we encountered three types of errors, as described in Table 5.

A record of the errors was maintained in an excel sheet, where 22 segments were listed along the columns and the recorded samples were listed along the rows. If a segment had any of the above errors in any segment, then* i*,* m*, or* d* were mentioned under that segment. The next step was the correction of these errors by updating the start and end times of the segments, because these errors occur due to incorrect labeling of these two times. After updating the time, the erroneous segments were extracted again by using updated time information. All tasks associated with the segmentation of the AVPD are presented and described in Table 6.

## 3. Evaluation of the AVPD

To evaluate the AVPD, various experiments for detection and classification of voice disorders were performed by implementing an automatic assessment system. The same experiments were performed for the Massachusetts Eye and Ear Infirmary (MEEI) to compare its results with those of the AVPD. The automatic assessment system is comprised of two main modules: the first module is the extraction of speech features, and the second module is pattern matching, which is implemented by using different machine learning techniques.

### 3.1. Feature Extraction Techniques

Many speech features extraction algorithms, MFCC, LPC, LPCC, PLP, RASTA-PLP, and MDVP, were implemented in this module of the automatic assessment system. Before the extraction of features, the speech signal was divided into frames of 20 milliseconds, which made the analysis easy because speech changes quickly over time. The MFCC mimics the human auditory perception, while the LPC and the LPCC mimic the human speech production system. The PLP and the RASTA-PLP simulate, to some extent, both the auditory and the production mechanisms. In the MFCC [17, 18], the time-domain speech signal was converted into a frequency-domain signal, which was filtered by applying a set of band-pass filters. The center frequencies of the filters were spaced on a Mel-scale and the bandwidths corresponded to the critical bandwidths of the human auditory system. The Mel-scale filter is given by (1), where *f* is frequency in Hz and *m* represents the corresponding frequency in Mel-scale. In this study, 29 Mel-scale filters are used. Later, a discrete cosine transform was applied to the filtered outputs to compress and decorrelate them.

(1) $$\begin{array}{c} m=2595\;\log_{10}\left(\;1+\frac{f}{700}\;\right). \end{array}$$

During extraction of the LPC features, the Linear Prediction (LP) analysis was performed. The LP analysis applies reverse filtering on speech signals to remove the effects of formants in order to estimate the source signal [29]. For LP analysis of order* P*, the current sample of a source signal can be estimated by using* P* previous samples by using

(2) $$\begin{array}{c} x_{r}^{\prime }=\overset{P}{\underset{i=1}{\sum }}a_{i}x_{r-i}, \end{array}$$

where, *x*_1_, *x*_2_, *x*_3_,…, *x*_*r*_ are samples of original speech signal and *a*_*i*_'s represent the required LPC features. To get accurate LPC features, it is necessary to reduce the error* E* between the current and estimated sample. This can be done by substituting the first-order derivative of* E* equal to zero and solve the resulting equations by using the Levinson-Durbin algorithm [30]. Moreover, the LPCC features are calculated by using the recursive relation [31] given in (3), where *σ*^2^ is the gain in LP analysis,* P* is the order of the LP analysis, *a*_*n*_ are LPC features, and *c*_*n*_ are obtained LPCC features. In this study, we performed LP analysis with *P* = 11.

(3) c1=ln⁡σ2cn=an+∑k=1n−1knckan−k,1<n≤Pcn=∑k=1n−1knckan−k,n>P.

The extraction of PLP features depends on three psychoacoustic principles of hearing [32]: (1) critical bandwidth, (2) equal-loudness hearing curve, and (3) intensity loudness power law of hearing. The critical bandwidths are computed by applying the Bark-scale proposed by Zwicker [33]. The sensitivity of the human auditory mechanism to different frequencies is different at the same sound intensity. Therefore, each critical band is multiplied with the equal-loudness weight. The weight for the *j*th critical band is computed as

(4) $$\begin{array}{c} W_{j}=\frac{{f_{j}}^{2}\times \left({f_{j}}^{2}+1.44\times 10^{6}\right)}{\left({f_{j}}^{2}+1.6\times 10^{5}\right)\times \left({f_{j}}^{2}+9.61\times 10^{6}\right)}. \end{array}$$

The center frequency of the *j*th critical band is represented by *f*_*j*_ in (4). Furthermore, the intensity loudness power law of hearing is used to simulate the nonlinear relationship between the intensity of sound and perceived loudness [34]. The extraction process of RASTA-PLP is the same as PLP, except that the RASTA filter given by (5) is applied after the critical bandwidth phenomena to remove the effect of constant and slowly varying parts [22].

(5) $$\begin{array}{c} R\left(\;z\;\right)=z^{4}\times \frac{\left(0.2+0.1z^{-1}-0.1z^{-3}-0.2z^{-4}\right)}{1-0.94z^{-1}}. \end{array}$$

In all types of experiments, static as well as delta and delta-delta features were considered. The delta and delta-delta coefficients were computed by taking the first-order and second-order derivatives of static features, respectively. The derivative was calculated by taking the linear regression with a window size of five elements. All experiments for MFCC, LPCC, and RASTA-PLP were conducted using 12 features (static), 24 features (12 static and 12 delta), and 36 features (12 static, 12 delta, and 12 delta-delta). For LPC and PLP, all experiments were performed by using only 12 static features.

In addition, 22 acoustic parameters were also extracted from each normal and pathological sample. These 22 speech samples are defined in Table 1 of [35], and they were extracted by using MDVP software [5]. This software is used frequently for the objective assessment of voice disorders in clinics.

### 3.2. Pattern Matching

The computed features are multidimensional and their interpretation is not easy for the human mind. Therefore, a pattern-matching phase becomes important in such situations in order to determine the trend in the data [36]. In this study, the pattern matching was performed by using different machine learning techniques, which performed better than statistical approaches in different areas [37, 38]. Machine learning techniques do not make strict assumptions about the data but instead learn to represent complex relationships in a data-driven manner [39].

In this module, various machine learning techniques (e.g., SVM [26], VQ [27], GMM [23], and HMM [24, 25]) were implemented for automatic detection and classification of voice disorders. SVM was implemented with linear and RBF kernels, GMM was implemented using 2, 4, 8, 16, and 32 mixtures, VQ used 2, 4, 8, 16, and 32 codebooks to generate acoustic models, and HMM was applied by using five states with 2, 4, and 6 mixtures in each state.

### 3.3. Detection and Classification Results for the AVPD

Experiments for detection determine whether an unknown test sample is normal or disordered. It is a two-class problem: the first class consists of all normal samples, and the second class contains samples of all types of disorder. During the classification of disorders, the objective is to determine the type of voice disorder. The classification of voice disorders is a many class problem, and the number of classes depends upon the number of types of voice disorder. The number of classes in this study was five because the AVPD has five types of vocal fold disorders: sulcus, nodules, cysts, paralysis, and polyps.

To be consistent, all voice samples of the MEEI and the AVPD are downsampled to 25 KHz, and each speech signal was divided into a frame of 20 milliseconds with 50% overlapping the previous frame. To avoid bias in the training and testing samples, all experiments were performed using a fivefold cross validation approach. In this approach, all samples were divided into five disjointed testing sets. Each time one of the sets was used to test the system, the remaining four were used to train the system. The accuracy of detection and classification of voice disorders for the AVPD with the sustained vowel /AH/ and the running speech “Al-Fateha” are listed in Table 7. In all experiments, the accuracy for detection and classification was calculated by using

(6) Accuracy%=Total  Correctly  Detected  SamplesTotal  Number  of  Samples×100.

Only the overall best accuracies (%) of voice disorder detection and classification for all types of feature extraction and machine learning techniques are presented in Table 7. For instance, the best detection accuracies for 12, 24, and 36 MFCC features are 73.59% with two Gaussian mixtures, 72.78% with four Gaussian mixtures, and 74.42% also with four Gaussian mixtures, respectively. However, only the overall best accuracy of 74.4% is mentioned in Table 7, and it is averaged over fivefold. The highest detection rate for a sustained vowel with MFCC is obtained by using SVM, which is 76.5%. However, among all feature extraction techniques, the maximum obtained detection rate is 79.5%. This maximum detection rate is achieved with MDVP by using SVM. In Table 7, “—” represents that experiments are not applicable here. For running speech, the maximum detection rate is 81.6%, which is obtained by using PLP and HMM. Similarly, the maximum accuracy for classification of voice disorder is 92.9% for sustained vowels and obtained with RASTA-PLP by using SVM. Furthermore, in the case of running speech, the maximum classification accuracy is 92.72%, which is obtained with RASTA-PLP and HMM.

### 3.4. Detection and Classification Results for the MEEI

All experiments performed for the AVPD were also performed with the MEEI database in order to make a comparison between the results. The experimental setup for the MEEI database is the same as the one used for the AVPD. The MEEI database was recorded by the Massachusetts Eye and Ear Infirmary voice and speech laboratory [40], and the language of the database is English. A subset of the database that has been used in a number of studies was considered for the experiments in this study [9, 36, 41–44]. The subset contained 53 normal subjects and 173 samples of disordered subjects suffering from adductor spasmodic dysphonia, nodules, keratosis, polyps, and paralysis. The detection and classification accuracies for the MEEI database are presented in Table 8. The maximum obtained detection accuracy for the MEEI database with the sustained vowel is 93.6%, which is obtained by using MFCC and RASTA-PLP when used with SVM. The maximum accuracy for running speech is 98.7%, obtained by using LPC and GMM. Similarly, for the classification of disorders, the maximum obtained accuracy with the sustained vowel is 98.2%, achieved with LPCC and PLP with VQ. The classification of disorders with running speech obtained an accuracy of 97.3% by using SVM with all types of speech features.

## 4. Discussion

Various steps for development of the AVPD, from recording protocol to the statistics, are presented in this study. Ethnicity influences the voice characteristics of people, as concluded by Walton and Orlikoff [1] and Malki et al. [4]. Therefore, the development of the AVPD was a good initiative, and it will contribute in the area of pathology assessment, especially in the Arab region. The AVPD is compared with the German voice disorder database (SVD) and an English voice disorder database (MEEI) by different aspects in Table 9. The SVD and MEEI databases are only two publicly available voice disorder databases.

During the development of the AVPD, different shortcomings of the SVD and MEEI databases were avoided. For instance, only the phonation part of the vowels is available in the SVD and MEEI databases, whereas De Krom [45] suggested that the complete recording of a vowel, including onset and offset parts, provides more acoustic information than only sustained phonation. Another drawback of a sustained phonation is a loss of information of the signal-to-noise ratio because a complete recording, including silence at the start and end of the recording, is necessary for its computation. In addition, the perpetual severity plays a very important role in pathology assessment, and it is not available in either the SVD or MEEI databases. In an automatic disorder detection system, a confusion matrix provides the information about truly and falsely classified subjects. In that matrix, the perceptual severity can determine the reason for misclassification. Automatic systems are sometimes unable to differentiate between normal and mildly severe pathological subjects. This is the reason why perceptual severity is also considered in the AVPD and rated over the scale of 1 to 3, where 3 represents a voice disorder with a high severity. Furthermore, the normal subjects in the AVPD are recorded after the clinical evaluation under the same condition as those used for the pathological subjects. In the MEEI database, the normal subjects are not clinically evaluated, although they do not have any history of voice complication [44]. In the SVD database, no such information is mentioned.

The AVPD has a balance between the number of normal and pathological subjects. Normal subjects are 51% of the total subjects in the AVPD. On the other hand, the percentage of normal subjects in the MEEI and SVD databases are 7% and 33%, respectively. The number of normal subjects in the MEEI database compared with pathological subjects is alarming. The numbers of normal and pathological samples in the MEEI database are 7% and 93%, respectively. As a result, an automatic system for disorder detection based on the MEEI database may be biased and cannot provide reliable results. For example, Dibazar et al. [46] obtained a classification accuracy of 65.26% when MFCC features are used with the nearest mean classifier. The numbers of normal and pathological samples used in the study are 53 and 657, respectively, taken from MEEI database. The interpretation of the results (accuracy of 65.26%) becomes difficult when data are unbalanced, because it cannot be determined how many normal and pathological samples are detected correctly by the system. One of the many possibilities may be that specificity is 0% and sensitivity is 70.47%. Another possibility may be that specificity is 100% and sensitivity is 62.40%. Specificity is a ratio between correctly detected normal samples and the total number of normal samples, and sensitivity is a ratio between correctly detected pathological samples and the total number of pathological samples [47]. The problem occurs due to imbalanced normal and pathological data. Therefore, Arjmandi et al. used 50 normal and 50 pathological samples to establish a balance between normal and pathological subjects in the study [35]. Unfortunately, this significantly limited the total sample number, which may have affected the reliability of results obtained in the study.

Unlike the MEEI database, it is assured that all normal and pathological samples are recorded at a unique sampling frequency in the AVPD. It is important because Deliyski et al. concluded that sampling frequency influenced the accuracy and reliability of acoustic analysis [48]. In addition, the MEEI database contains one vowel, whereas the AVPD records three vowels. Although the SVD also records three vowels, they are recorded only once. In the AVPD, the three vowels are recorded with a repetition, as some studies recommended that more than one sample of the same vowel helps to model the intraspeaker variability [49, 50]. Another important characteristic of the AVPD is the total length of the recorded sample, which is 60 seconds, as described in Table 1. All recorded text in the AVPD is of the same length for normal as well as disordered subjects. In the MEEI database, the recording times for normal and pathological subjects are different. Moreover, the duration of connected speech (a sentence) in the SVD database is only 2 seconds, which is too short and not sufficient to develop an automatic detection system based on connected speech. Furthermore, a text-independent system is not possible to build with the SVD database. The average length of the running speech (Al-Fateha) in the AVPD is 18 seconds, and it consists of seven sentences. Al-Fateha is segmented into two parts, as described in Section 2.5, so that it may be used to develop text-independent systems.

Another unique aspect of the AVPD, other than the perceptual severity, is the recording of the isolated words. The AVPD contains 14 isolated words: Arabic digits (zero to ten) and three common words. The common words were recorded to introduce the missing Arabic letters. In this way, the AVPD contains each and every Arabic letter. Due to the isolated words, the AVPD can be used to develop different speech recognition applications for dysphonic patients, which are not possible to develop by using the SVD and MEEI databases. The development of a speech recognition system that can detect how accurate the speech of a voice-disordered patient is or has improved after treatment may have important prognostic value throughout the management of voice disorders.

**Figure 3:**
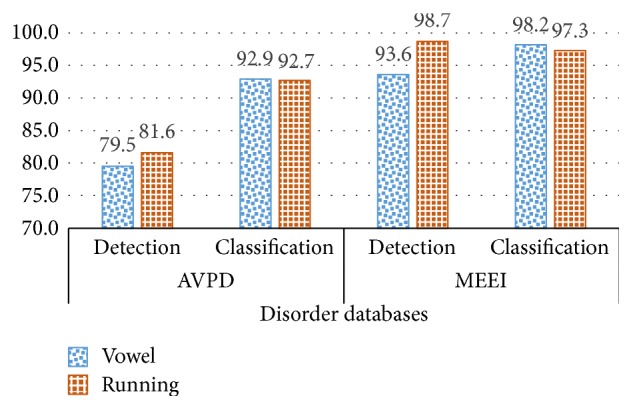
Comparison of detection and classification accuracy for the AVPD and MEEI databases.

Different automatic detection systems are implemented to evaluate the AVPD, and the obtained results are compared with the results of the MEEI database. Automatic detection systems have extracted the following speech features: MDVP, MFCC, LPCC, RASTA-PLP, LPC, and PLP. For every type of feature, each of the following machine learning algorithms is implemented: SVM, GMM, HMM, and VQ. All accuracies of automatic detection systems for the AVPD and the MEEI database are presented in Tables 7 and 8. A comparison of the highest accuracies for the detection and classification of the AVPD and MEEI databases is depicted in Figure 3. It can be observed from Figure 3 that the highest accuracy for detection with the sustained vowel is 79.5% for the AVPD and 93.6% for the MEEI database.

Similarly, the maximum accuracy for detection with running speech is 81.6% for the AVPD and 98.7% for the MEEI database. There is a significant difference between accuracies of the MEEI database and the AVPD, 14.1% for sustained vowels and 17.1% for running speech. The same kind of trend for accuracy is observed in the study by [51], in which the results of the MEEI database were compared with the results of a Spanish database (UPM). A difference of 20% was observed between accuracies. In another study [52], the result obtained with the sustained vowel /a/ for the MEEI database was around 95%, while that for the SVD was approximately 80%. Again, a significant difference of 15% was observed. The reason for the difference might be the recording environments of the MEEI database, as [53] mentions that* “Normal and pathological voices were recorded at different locations (Kay Elemetrics and MEEI Voice and Speech Lab., respectively), assumedly under the same acoustic conditions, but there is no guarantee that this fact has no influence on an automatic detection system.”*

## 5. Conclusion

Design, development, and evaluation of the AVPD have been presented in this study. The AVPD could be a key factor in the advances of voice pathology assessment for the Arab region. Dysphonic patients suffering from five different types of organic voice disorders (cysts, nodules, polyps, paralysis, and sulcus) were included in the database. The database contains repeated vowels, a running speech, Arabic digits, and some common words. The rating of perceptual severity of the voice disorders and recording of isolated words are the unique aspects of the AVPD. All subjects, including patients and normal persons, were recorded after clinical evaluation. Baseline results of the AVPD were provided by using various types of speech features with a number of machine learning algorithms. The accuracy for detection and classification of voice disorders was computed for the sustained vowels as well as for the running speech. The obtained results were compared with the English voice disorder database (MEEI), and the classification results of the two were comparable, although a significant difference was observed in the case of disorder detection. The detection results of the MEEI database also differ significantly from the German voice disorder database (SVD). The reason might be the different recording environments of the MEEI database for normal and pathological subjects. Therefore, different shortcomings of the SVD and MEEI databases were taken into account before recording the AVPD.

## Supplementary Material

cyt-m-55-n-360527-kam-3-p.wav This is recording of a 55 years old male subject who is suffering from cyst. The subject has no habit of smoking, perceptual severity is 3 and recording is done before surgery .nor-m-23-n-90021-kac-0-p.wav This is a recording of a 23 years old male subject who is normal. The subject has no habit of smoking. As subject is normal, therefore, severity is 0 and no surgery is performed.prl-f-34-n-569624-kac-2-p.wav This is a recording of a 34 years old female subject who is suffering from paralysis. The subject has no habit of smoking, perceptual severity is 2 and recording is done before surgery .sls-m-20-y-545716-kac-1-p.wav This is recording of a 20 years old male subject who is suffering from sulcus. The subject has no habit of smoking, perceptual severity is 1 and recording is done before surgery.

## Figures and Tables

**Table 1:** Arabic digits with international phonetic alphabets (IPAs) and English translation.

Arabic digits	English translation	IPAs of Arabic digits
صفر	Zero	/*ṣ*/, /i/, /f/, /r/
واحد	One	/w/, /a/, /*ħ*/, /i/, /d/
أثنين	Two	/a/, /th/, /n/, /a/, /y/, /n/
ثلاثة	Three	/th/, /a/, /l/, /$\overset{-}{a}$/, /th/, /a/
أربعة	Four	/a/, /r/, /b/, /ʕ/, /a/
خمسة	Five	/kh/, /a/, /m/, /s/, /a/
ستة	Six	/s/, /i/, /t/, /t/, /a/
سبعة	Seven	/s/, /a/, /b/, /ʕ/, /a/
ثمانية	Eight	/th/, /a/, /m/, /$\overset{-}{a}$/, /n/, /y/, /a/
تسعة	Nine	/t/, /i/, /s/, /ʕ/, /a/
عشرة	Ten	/ʕ/, /a/, /*ʃ*/, /a/, /r/, /a/
—		

**Table 2:** Common words with IPAs and English translation.

Common words	English translation	IPAs of common words
ظرف	Envelope	/z/, /a/, /r/, /f/
غزال	Deer	/*ɣ*/, /a/, /z/, /a/, /l/
جمل	Camel	/j/, /a/, /m/, /a/, /l/

**Table 3:** Text from Al-Fateha with English translation.

English translation	Al-Fateha	Sentence number
Praise be to God, Lord of all the worlds	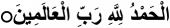	1

The Compassionate, the Merciful	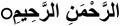	2

Ruler on the Day of Reckoning	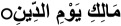	3

You alone do we worship, and You alone do we ask for help	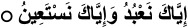	4

Guide us on the straight path	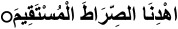	5

The path of those who have received your grace	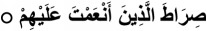	6

Not the path of those who have brought down wrath, nor of those who wander astray	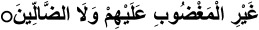	7

**Table 4:** Number of occurrences of each Arabic letter in the recorded text.

Letters	Number of occurrences
ا‎	30
ب‎	5
ت‎	5
ث‎	4
ج‎	1
ح‎	4
خ‎	1
د‎	5
ذ‎	1
ر‎	10
ز‎	1
س‎	6
ش‎	1
ص‎	3
ض‎	2
ط‎	2
ظ‎	1
ع‎	10
غ‎	3
ف‎	2
ق‎	1
ك‎	3
ل‎	21
م‎	15
ن‎	13
ه‎	4
و‎	5
ي‎	14

**Table 5:** Description of errors encountered during the verification process.

Errors in the segments	Abbreviation	Description	Examples
Incomplete	i	When some part of the extracted text is missing at the start or end	(a) “d” is missing in* wahid*(b) “w” is missing in* wahid*(c) Both “w” and “d” are missing
More	m	When a segment contains some part of the next or previous segment	(a) Segment of* Sifar* also contains “w” of next segment *wahid* (b) Segment of *Ithnayn* also contains “d” of previous segment *wahid*

Different	d	When the text in a segment is other than the expected one	Segment contains* wahid* instead of *sifar*

**Table 6:** Tasks for the AVPD.

Number	Tasks	Description
Task 1	Time labeling	Start and end times of the recorded vowels, digits, Al-Fateha, and common words

Task 2	Extraction	By using start and end times, the recorded vowels, digits, Al-Fateha, and common words are extracted and stored in a new *wav* file

Task 3	Verification	Verification of the extracted vowels, digits, Al-Fateha, and common words

*When errors are found during verification process (Task 3), continue with Tasks 4 and 5*		

Task 4	Repeat time labeling	Update start and end time of the erroneous segments

Task 5	Repeat extraction	Extract the segments again using updated time

**Table 7:** Overall best accuracies (%) for sustained vowels and running speech by using the AVPD.

Features	Experiments	SVM		GMM		VQ		HMM	
		*/AH/*	*Al-Fateha*	*/AH/*	*Al-Fateha*	*/AH/*	*Al-Fateha*	*/AH/*	*Al-Fateha*
MFCC	Detection	76.5^*∗*^	77.4	74.4	77.1	70.3	71.1	71.6	78.1^*▯*^
	Classification	89.2^*α*^	89.2	88.9	89.5	75.3	81.6	88.7	90.9^*β*^

LPCC	Detection	60.1	76.5	54.5	76.7^*▯*^	70.3	75.9	73.5^*∗*^	71.5
	Classification	67.6	84.7	75.4	86.0^*β*^	75.5^*α*^	77.9	59.0	86.0^*β*^

RASTA-PLP	Detection	77.0^*∗*^	76.7	72.8	74.5	67.1	75.0	66.3	79.0^*▯*^
	Classification	92.9^*α*^	90.2	91.3	91.2	88.9	90.3	88.7	92.7^*β*^

LPC	Detection	62.3	71.6	53.7	71.9^*▯*^	70.7	71.5	71.4^*∗*^	62.3
	Classification	66.3	82.4^*β*^	74.6	79.7	78.6	75.3	85.9^*α*^	75.9

PLP	Detection	75.8^*∗*^	79.1	73.2	78.5	72.0	78.1	73.6	81.6^*▯*^
	Classification	91.5^*α*^	90.1	88.9	91.2^*β*^	79.4	77.2	88.7	85.8

MDVP	Detection	79.5^*∗*^	—	69.8	—	64.8	—	—	—
	Classification	82.3^*α*^	—	—	—	—	—	—	—

^*∗*^The best detection rate for sustained vowels. ^*▯*^The best detection rate for running speech. ^*α*^The best classification rate for sustained vowels. ^*β*^The best classification rate for running speech.

**Table 8:** Overall best accuracies (%) for sustained vowels and running speech by using the MEEI database.

Features	Experiments	SVM		GMM		VQ		HMM	
		*/AH/*	*Rainbow*	*/AH/*	*Rainbow*	*/AH/*	*Rainbow*	*/AH/*	*Rainbow*
MFCC	Detection	93.6^*∗*^	97.4	91.6	97.3	90.3	96.0	88.9	98.3^*▯*^
	Classification	95.4	97.3^*β*^	97.3^*α*^	97.3	96.3	97.3	87.5	88.9

LPCC	Detection	91.0^*∗*^	97.9	90.7	96.4	83.2	97.8	87.6	98.2^*▯*^
	Classification	95.4	97.3^*β*^	97.3	97.3	98.2^*α*^	97.3	87.5	97.3

RASTA-PLP	Detection	93.6^*∗*^	98.0	91.6	98.1^*▯*^	84.1	96.4	88.9	98.1
	Classification	95.5	97.3^*β*^	97.3^*α*^	97.3	97.3	96.3	85.2	84.6

LPC	Detection	82.9	96.0	83.2^*∗*^	98.7^*▯*^	78.3	97.3	80.1	96.3
	Classification	95.2	97.3^*β*^	97.3^*α*^	97.3	97.3	94.4	75.0	82.5

PLP	Detection	87.8	96.8	91.2^*∗*^	97.8^*▯*^	89.4	97.8^*▯*^	87.4	96.3
	Classification	95.0	97.3^*β*^	97.3	97.3	98.2^*α*^	94.4	61.1	84.6

MDVP	Detection	89.5^*∗*^	—	88.3	—	68.3	—	—	—
	Classification	88.9^*α*^	—	—	—	—	—	—	—

^*∗*^The best detection rate for sustained vowels. ^*▯*^The best detection rate for running speech. ^*α*^The best classification rate for sustained vowels. ^*β*^The best classification rate for running speech.

**Table 9:** Comparison of AVPD with two publicly available voice disorder databases.

Sr. number	Characteristics	MEEI	AVPD	SVD
(1)	Language	English	Arabic	German

(2)	Recording location	Massachusetts Eye & Ear Infirmary (MEEI) voice and speech laboratory, USA	Communication and Swallowing Disordered Unit, King Abdulaziz University Hospital, Saudi Arabia	Saarland University, Germany

(3)	Sampling frequency	Samples are recorded at different sampling frequencies (i) 10 kHz (ii) 25 kHz (iii) 50 kHz	All samples are recorded at same frequency (i) 48 kHz	All samples are recorded at same frequency (i) 50 kHz

(4)	Extension of recorded samples	Recorded samples are stored in .NSP format only	Recorded samples are stored in .wav and .nsp format	Recorded samples are stored in .wav and .nsp format

(5)	Recorded text	(i) Vowel /a/ (ii) Rainbow passage	(i) Vowel /a/ (ii) Vowel /i/ (iii) Vowel /u/ (iv) Al-Fateha (running speech) (v) Arabic digits (vi) Common words (All vowels are recorded with a repetition)	(i) Vowel /a/ (ii) Vowel /i/ (iii) Vowel /u/ (iv) A sentence

(6)	Recording of vowels	Only stable part of the phonation	Complete phonation including onset and offset parts	Only stable part of the phonation

(7)	Length of recorded samples	Normal (i) Vowel: 3 sec (ii) Rainbow: 12 sec Patient (i) Vowel: 1 sec (ii) Rainbow: 9 sec	(i) Vowel: 5 sec (ii) Al-Fateha: 18 sec (iii) Digits: 10 sec (iv) Words: 3 sec (The length of a complete recorded sample is 60 sec approx.)	Vowels: 1~3 sec Sentence: 2 sec

(8)	Ratio of normal and pathological subjects	Normal: 7% Pathological: 93%	Normal: 51% Pathological: 49%	Normal: 33% Pathological: 67%

(9)	Perceptual severity	*✗*	✓ Perceptual severity is rated on a scale of 1 (low) to 3 (high)	*✗*

(10)	Pathology types	Functional and organic	Organic	Functional and organic

(11)	Evaluation of normal subjects	*✗*	✓	No such information is available
